# Protective Effect of Thymosin β4 against Abdominal Aortic Ischemia–Reperfusion-Induced Acute Lung Injury in Rats

**DOI:** 10.3390/medicina55050187

**Published:** 2019-05-22

**Authors:** Onur M. Yaman, Ibrahim Guner, Gulcan Guntas, Osman Fuat Sonmez, Gamze Tanriverdi, Aris Cakiris, Ugur Aksu, Sibel Akyol, Elif Guzel, Hafize Uzun, Nermin Yelmen, Gulderen Sahin

**Affiliations:** 1 Department of Physiology, Cerrahpasa Medical Faculty, Istanbul University-Cerrahpasa, 34098 Istanbul, Turkey; monur.yaman@istanbul.edu.tr (O.M.Y.); guner@istanbul.edu.tr (I.G.); ofuats@gmail.com (O.F.S.); sakyol@istanbul.edu.tr (S.A.); nermink@istanbul.edu.tr (N.Y.); 2 Department of Nursery, School of Health, University of Kırklareli, 39060 Kırklareli, Turkey; gulcanguntas@klu.edu.tr; 3 Department of Histology and Embriology, Cerrahpasa Medical Faculty, Istanbul University-Cerrahpasa, 34098 Istanbul, Turkey; tgamze@istanbul.edu.tr (G.T.); elifguzelctf@yahoo.com (E.G.); 4 Institute of Health Sciences, Istanbul University, Genetics, 34098 Istanbul, Turkey; cikiris@yahoo.com; 5 Department of Biology, Science Faculty, Istanbul University, 34098 Istanbul, Turkey; uguraksu@istanbul.edu.tr; 6 Department of Biochemistry, Cerrahpasa Medical Faculty, Istanbul University-Cerrahpasa, 34098 Istanbul, Turkey; huzun59@hotmail.com

**Keywords:** thymosin beta 4, ischemia–reperfusion, lung injury, oxidative stress, inflammation

## Abstract

*Background and objectives*: Ischemia–reperfusion (IR) caused by infrarenal abdominal aorta cross-clamping is an important factor in the development of ischemia–reperfusion injury in various distant organs. *Materials and Methods:* We investigated potential antioxidant/anti-inflammatory effects of thymosin beta 4 (Tβ4) in a rat model of abdominal aortic surgery-induced IR. Tβ4 (10 mg/kg, intravenous (i.v.)) was administered to rats with IR (90-min ischemia, 180-min reperfusion) at two different periods. One group received Tβ4 1 h before ischemia, and the other received 15 min before the reperfusion period. *Results:* Results were compared to control and non-Tβ4-treated rats with IR. Serum, bronchoalveolar lavage fluid and lung tissue levels of oxidant parameters were higher, while antioxidant levels were lower in the IR group compared to control. IR also increased inflammatory cytokine levels. Tβ4 reverted these parameters in both Tβ4-treated groups compared to the untreated IR group. *Conclusions:* Since there is no statistical difference between the prescribed results of both Tβ4-treated groups, our study demonstrates that Tβ4 reduced lung oxidative stress and inflammation following IR and prevented lung tissue injury regardless of timing of administration.

## 1. Introduction 

Occlusion and reperfusion of the infrarenal abdominal aorta (IAA) occurs in humans during perivascular surgery [1] and when applied in experimental animal models [2,3]. This method is known to cause ischemia–reperfusion injury (IRI) in various distant organs, such as the kidney, heart, and lungs, which may lead to multiple organ dysfunction syndrome (MODS). A myriad of factors, such as increased oxidative stress, neutrophil infiltration, various pro-inflammatory cytokine activities, endothelial injury, and disruption of cell membrane ion transport mechanisms, all contribute to IRI [4].

Reactive oxygen species (ROS) have a key role in the development of ischemia-reperfusion-induced lung injury [5], with a crucial role in the sequence of events leading to lung failure [2]. ROS, derived from H_2_O_2_, NADPH (Nicotinamide adenine dinucleotide phosphate) oxidase, and other enzymatic reactions are toxic molecules that lead to tissue damage. In particular, the hydroxyl radical primarily reacts with the lipid membrane components of the cell, causing lipid peroxidation that may lead to increased permeability and cell lysis [5]. ROS mediates tissue injury by means of endothelial activation, cytokine secretion, and leukocyte activation. Activated leukocytes provoke local and systemic reperfusion inflammatory responses. Systemic inflammatory response syndrome (SIRS) is often seen in patients undergoing abdominal aortic aneurysm repair, is the first step in the progression of MODS, and is largely due to the activation of the inflammatory pathway mediated by cytokines [6]. 

During ischemia-reperfusion (IR) of the lungs, numerous pro- inflammatory cytokines, such as tumor necrosis factor (TNF-α), interleukin 1β (IL-1β), and interleukin 6 (IL-6), and transcription factors, including nuclear factor kappa B (NF-κB), are rapidly released. NF-κB upregulates pro-inflammatory cytokines, chemokines, apoptotic signals, and cell adhesion molecules [7]. 

Antioxidants significantly delay or inhibit the oxidation of a substrate, even at low concentrations. Endogen antioxidant enzymes, such as superoxide dismutase, glutathione reductase, catalase, and glutathione peroxidase, protect the cell against the harmful effects of ROS. Therefore, measurement of those enzymes indicates the magnitude of oxidative stress during IRI [2].

In this respect, compounds with antioxidant and anti-inflammatory properties, such as thymosin beta 4 (Tβ4), can be good candidates for the prevention and treatment of IR-mediated lung injury. Tβ4 was reported to have antioxidative [8,9], anti-inflammatory [9], anti-apoptotic, and regenerative properties [10]. Tβ4 was shown to be expressed in various normal tissues of rat, mice, and human [10,11]. Tβ4 is found in all cells except erythrocytes; however, since it has no secretion signal, damaged cells are held responsible for its presence in all body fluids, including saliva, tears, blood plasma, and wound exudates [12]. In addition, Tβ4 is considered to be well tolerated and safe in pharmacological and toxicological experimental studies performed on rats, dogs, monkeys [13], and humans [14]. Considering these characteristics, we planned our study with the thought in mind that Tβ4 may have a protective effect against acute lung injury induced by aortic IR. 

In the present study, we tested the hypothesis that the administration of Tβ4 (either before ischemia or before reperfusion periods) may have a potential effect in a rat model of abdominal aortic surgery-induced IR by suppressing both oxidant and inflammatory responses. We also aimed to determine which period would be the most beneficial for systemic administration of Tβ4. Therefore, we determined various oxidants levels, including lipid hydroperoxide (LOOH), malondialdehyde (MDA), and pro-oxidant/antioxidant balance (PAB). We also evaluated antioxidant systems, including superoxide dismutase (Cu-/Zn-SOD), glutathione (GSH), and ferric reducing antioxidant power (FRAP), pro-inflammatory cytokines (TNF-α, IL-1β, IL-6), and transcription factor NF-κB in serum, to evaluate the systemic inflammatory response, in both lung tissue and bronchoalveolar lavage fluid (BALF) samples, to determine the lung injury in rats that underwent 90 min of IAA occlusion and 180 min of reperfusion. We also examined histopathological changes in the lung tissue.

## 2. Materials and Methods 

### 2.1. Animals

All experiments in this study were approved and reviewed by the Animal Research Committee of the University of Istanbul on 31 May 2012 (2012/65). Care and handling of the animals was in accordance with the Helsinki Declaration of 1975, as revised in 2000. Animals were housed in individual cages in a temperature-controlled room (23 ± 1 °C) and a light–dark cycle-controlled environment (12 h) with free access to food and water. Experiments were performed on 32 Sprague-Dawley rats with an average body weight of 350 ± 5 g.

### 2.2. Experimental Design

Rats were randomly divided into four groups as control (sham-operated), IR (with saline), Tβ4+IR, and I+Tβ4+R groups (*n* = 8 per group). The control group underwent midline laparotomy and dissection of IAA without occlusion. The IR group underwent laparotomy, followed by clamping (occlusion) of the IAA for 90 min of ischemia and then 180 min of reperfusion. Rats received saline solution in both control and IR groups. The rats in the Tβ4+IR group received Tβ4 (10 mg/kg, intravenous (i.v.)) 1 h before ischemia. The rats in the I+Tβ4+R group received Tβ4 (10 mg/kg, i.v.) 15 min before reperfusion. We have modified the Tβ4 dose and dose range according to a previous study by Philip and Kleinman. We used a single dose of 10 mg/kg (i.v.), which produces nontoxic effects [13].

### 2.3. Surgical Procedure

The rats were anesthetized with pentobarbital sodium (60 mg/kg, intraperitoneal (i.p.)). After tracheotomy, the animals were allowed to breathe spontaneously. Body temperature was maintained at 37 ± 0.5 °C during the entire experiment. The skin was aseptically prepared, and a midline laparotomy was performed. Then, 10 mL of saline was instilled into the peritoneal cavity to prevent fluid loss. The abdominal aorta was exposed by gently deflecting the intestine loops to the left. After fine isolation of the infrarenal segment, an atraumatic microvascular clamp (vascu-statts II, midistraight 1001-532; Scanlan Int. St Paul, MN, USA) was placed on the aorta. The abdomen was closed right after clamping, and the surgical area was covered with a humidified gauze compress throughout the entire experiment to prevent drying. The microvascular clamp on the infrarenal abdominal aorta (IAA) was removed after 90 min, and the reperfusion period was for 180 min. Before induction of ischemia, each animal received 50 U/kg (total volume 500 μL) heparin (Nevparin; Mustafa Nevzat Drug Company, Istanbul, Turkey) in saline intravenously via a lateral tail vein injection. Aortic occlusion and reperfusion were confirmed by the loss and reappearance of satisfactory pulsation in the distal aorta. The changes in the recording of systemic arterial blood pressures also confirmed this procedure. Bronchoalveolar lavage fluid (BALF) and blood samples for serum/plasma were obtained right after termination of the reperfusion period. At the end of the experiment, the animals were euthanized by deep anesthesia (pentobarbital sodium, 150 mg/kg, i.v. tail vein). Then, lungs were removed immediately and washed in 0.9% NaCl. The vertical outer half of the right lung was stored in formalin for histologic evaluation. The lower lobe of the right lung was used to determine the ratio of the lung wet to dry (W/D) weight. The remaining portions of the lungs and serum/plasma samples were stored at −80 °C until assayed for levels of oxidative stress and inflammation. LOOH, MDA, GSH, SOD, PAB, FRAP, TNF-α, IL-6, IL-1β, and NF-κB levels were measured spectrophotometrically or with ELISA in all samples.

### 2.4. Chemicals

Synthetic Tβ4 was provided as a gift from RegeneRx Biopharmaceuticals, Rockville, MD, USA. Synthetic Tβ4 was stored at −20 °C; all other reagents were stored at 4 °C. The reagents were equilibrated at room temperature before use.

### 2.5. Bronchoalveolar Lavage Fluid (BALF)

The left main bronchus was cannulated and secured. Saline (5 mL) was then injected as three aliquots of 5 mL each. Each aliquot was injected quickly and then withdrawn slowly three times to obtain the BALF specimen. Fluid recovery was routinely 90% or greater. This fluid was centrifuged (1500 rpm for 8 min at 4 °C). The supernatant was stored at −80 °C until assayed for biochemical evaluations.

### 2.6. Biochemical Analyses

#### 2.6.1. Determination of Protein Concentration

The bicinchoninic acid assay (BCA) was performed using Thermo Scientific Pierce BCA Protein Assay Kit (Life Technologies Cat No: 23227) to measure (absorbance at 562 (A562) nm) total protein concentration compared to a protein standard. Bovine serum albumin (BSA) was used as a protein standard. Each sample in the plate was measured in BIO-TEK ELx800 Universal Microplate Reader, and concentrations were calculated using a standard curve. The samples were stored at −80 °C for later use.

#### 2.6.2. Oxidative Stress Parameters Measurements in Serum, BALF, and Lung Tissue Samples

Lipoperoxidation of all samples was ascertained by the formation of malondialdehyde (MDA), which was estimated using the modified thiobarbituric acid method [15]. Lipid hydroperoxide (LOOH) levels were determined spectrophotometrically according to the method of ferrous oxidation with xylenol orange version 2 (FOX2) [16].

The non-enzymatic antioxidant level of samples was evaluated with the ferric reducing antioxidant power (FRAP) assay and was performed according to the protocol of Benzie and Strain [17]. The pro-oxidant/antioxidant balance (PAB) assay was performed according to the method of Alamdari et al. [18]. The values of the PAB are expressed in arbitrary Hamidi–Koliakos (HK) units, which represent the percentage of hydrogen peroxide in the standard solution. 

The enzymatic antioxidant defense system of samples was evaluated with both Cu-/Zn-SOD activities and glutathione levels. The Cu-/Zn-SOD was determined using the method of Sun et al. [19] via inhibition of nitroblue tetrazolium (NBT) reduction with xanthine/xanthine oxidase used as a superoxide generator. One unit of Cu-/Zn-SOD was defined as the amount of protein that inhibits the rate of NBT reduction by 50%. The method of Beutler et al. [20]. was used for the determination of glutathione (GSH) levels. The GSH concentration was calculated with a molar absorption coefficient (ε) = 1.36 × 10^−4^ M^−1^∙cm^−1^ at a wavelength (λ) = 412 nm, and a standard curve was plotted at 540 nm using a spectrophotometer. All measurements related to oxidative stress were done as clearly stated in our previous studies [2,3,21]. The other biochemical parameters were measured by routine methods with commercial kits.

#### 2.6.3. Measurement of Inflammatory Cytokines and Nuclear Factor Kappa B

The levels of TNF-α, IL-1β, and IL-6 were determined by ELISA using commercially available kits from eBioscience (San Diego, CA, USA). The level of nuclear factor kappa B (NF-κB) was determined by ELISA using a commercially available kit from Abcam (Cambridge, MA, USA). All samples were measured in duplicate using the protocol provided by the manufacturer.

### 2.7. Histological Evaluation

For histological evaluation, lung tissues were dissected, fixed in 10% neutral formalin, embedded in paraffin wax, and then cut into 5-µm-thick sections. The sections were placed on slides, then deparaffinized in xylene, and rehydrated in graded alcohol. Slides were stained with hematoxylin and eosin (H&E) for basic histological evaluation and for detecting the general tissue morphology using standard protocols. The slides were examined and photographed under a light microscope (Olympus BX61, Tokyo, Japan). Two independent histologists assessed and scored the tissue injuries in a blinded fashion according to a scoring system modified from Kandilci et al. [22] and Pirat et al. [23]. The scoring was graded as follows: score “0 (null)” indicates 0% involvement, score “1” indicates 1–25% involvement, score “2” indicates 26–50% involvement, score “3” indicates 51–75% involvement, and score “4” indicates 75–100% involvement. Five parameters (interstitial edema/infiltration, intra-alveolar edema/infiltration, intra-alveolar hemorrhage, capillary congestion, and airway epithelial cell damage) were evaluated for lung injury, and the total lung injury score was calculated.

### 2.8. Statistical Analysis

Values are reported as means ± standard error of the mean (SEM). Statistical analysis was performed with GraphPad Prism version 5.0 for Windows (GraphPad Software v5.0, San Diego, CA, USA). One-way ANOVA analysis was used for comparisons; post hoc analyses were used with Tukey’s post hoc test when *p* < 0.05. For all analyses, *p* < 0.05 was considered significant.

## 3. Results 

Experiments were performed on 32 Sprague-Dawley rats randomly divided into four groups as control (sham-operated), IR (with saline), Tβ4+IR, and I+Tβ4+R groups (*n* = 8 per group).

### 3.1. Alterations of W/D (Wet to Dry) Weight Analysis

W/D ratio levels of the lung were as follows: control group, 3.23 ± 0.14; IR group, 5.44 ± 0.27; Tβ4 + IR group, 3.94 ± 0.32; and I+Tβ4+R group, 3.42 ± 0.29. The lung W/D ratio was significantly increased in the IR group compared with control (*p* < 0.001). Administration of Tβ4 markedly reduced the lung W/D ratio in both Tβ4+IR and I+Tβ4+R groups (*p* < 0.01 and *p* < 0.001, respectively vs. IR). 

### 3.2. Changes of Oxidative Stress Parameters in Rats

Occlusion and reperfusion of infrarenal abdominal aorta significantly increased LOOH, MDA, and PAB levels and significantly decreased SOD and FRAP levels in serum, BALF, and lung tissue samples compared to control group animals. Administration of Tβ4 before ischemia and before reperfusion periods significantly decreased LOOH, MDA, and PAB levels while significantly increasing SOD and FRAP levels in all available samples (Table 1).

### 3.3. Changes of Inflammatory Cytokines and NF-κB in Rats

Serum, BALF and lung tissue TNF-α, IL-1β and IL-6 levels were increased in aortic IR induced lung injury and the increase of NF-κB in all samples with IR are parallel to the increases of those pro-inflammatory cytokines. Tβ4 administration in both treated groups led to a normalization in both inflammatory cytokines and NF-κB levels (Table 2).

### 3.4. Changes in Lung Histology

**Figure 1:**
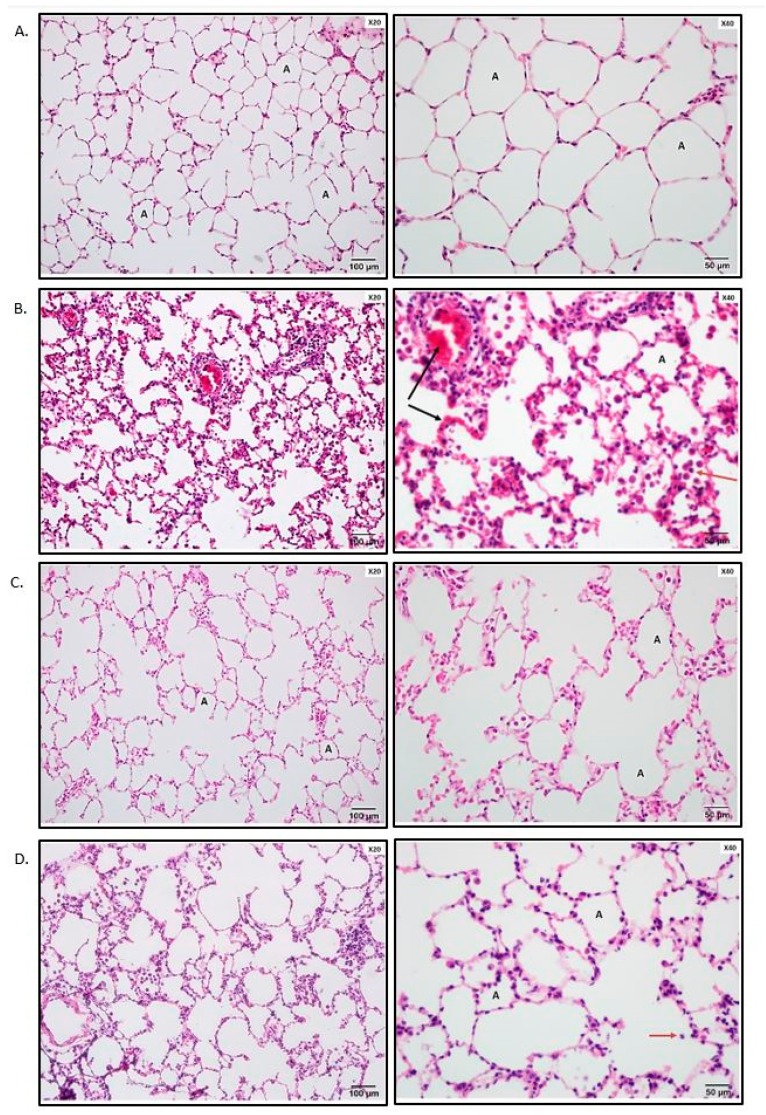

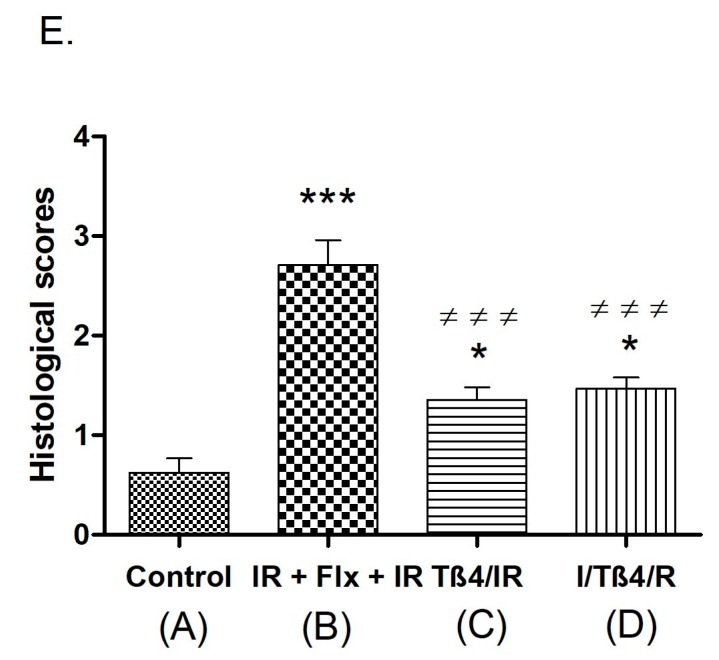
Photomicrographs of lung samples and histological scoring of lung tissue. * *p* < 0.05, *** *p* < 0.001 vs. control; ≠≠≠ *p* < 0.001 vs. ischemia–reperfusion (IR). (**A**) Lung samples of the control group were stained with hematoxylin and eosin (H&E). The control group shows minimal negligible histological alterations in lung tissue. The alveolar walls are very thin, and the majority of the alveoli contain no cells; A: alveolus. (**B**) Representative photomicrographs of lung samples of the IR group stained with hematoxylin and eosin. The IR group shows moderate lung ischemia–reperfusion injury with marked blood vessel congestion (black arrow) and leukocytic infiltration of the alveolar lumen (red arrow); A: alveolus. (**C**) Representative photomicrographs of lung samples of thymosin beta 4 (Tβ4)/IR stained with hematoxylin and eosin. The image demonstrates the improvement of the lung histopathology regarding the lung ischemia–reperfusion injury. (**D**) Representative photomicrographs of lung samples of I/Tβ4/R stained with hematoxylin and eosin. The I/Tβ4/R group, similar to the Tβ4/IR group, has less damage than the IR group; red arrow: infiltrative cells in the lumen of the alveoli. (**E**) Histological scoring of lung tissue.

The lungs of the sham-operated rats had minimal histological changes (Figure 1A). IR resulted in moderate lung damage, including capillary congestion, interstitial edema and infiltration, and intra-alveolar hemorrhage. The alveolar spaces were filled with mononuclear and neutrophilic infiltrates. Interstitial edema and inflammatory cell infiltration, together with capillary congestion, were noted to cause thickening and destruction of the alveolar walls in some areas (Figure 1B). The alveoli seem to be reduced in volume, and compensatory dilatation of the alveoli and alveolar sacs was seen in different neighboring regions in some specimens. The total lung injury score of the IR group was significantly higher when compared to that in the control group (*p* < 0.01) (Figure 1E). The injured areas exhibited heterogeneity throughout the specimens, with the pathological changes mentioned above interspersed with some areas showing only minimal changes. There was also swelling and vacuolization of the bronchiolar epithelium, and there was evidence of denuding in significant portions of the epithelium.

In both Tβ4+IR and I+Tβ4+R groups, the total lung injury score was significantly lower when compared to that in the IR group (*p* < 0.01) (Figure 1E). Animals in groups Tβ4+IR and I+Tβ4+R had significantly less lung damage than that in the IR group (*p* < 0.01) (Figure 1C,D). Some areas in some sections showed thickening of the interalveolar septum due to capillary dilatation and stasis and interalveolar infiltration, but it was less prominent than that in the IR group. The bronchiolar epithelium was partly desquamated. There was no statistically significant difference between the two treatment protocols regarding the total lung injury score (Figure 1E).

## 4. Discussion 

In the present study, we found that occlusion (90 min) and reperfusion (180 min) of infrarenal abdominal aorta (IAA)-induced IR significantly increased LOOH, MDA, and PAB levels and significantly decreased Cu-/Zn-SOD and FRAP levels in serum, BALF, and lung tissue samples. In addition to the oxidative stress-related biomarkers in all samples, pro-inflammatory cytokines including TNF-α, IL-6, IL-1β, and NF-κB were excessively increased. These results indicate that the activation of systemic ROS and inflammatory mediators triggers a sequence of events leading to acute lung injury (ALI). To the best of our knowledge, this is the first study to show that Tβ4 administration (before ischemia or before reperfusion) was able to improve IR-mediated ALI. Tβ4 achieved that goal by reducing oxidative stress, increasing antioxidant enzymes, reducing inflammation, and preventing cellular injury. These findings were supported by histological changes observed in lung tissue samples in all groups.

Recent studies suggested that oxidative stress [2], inflammation [24], cell necrosis, and apoptosis [4] may be held responsible for the development of acute lung injury all together. The lung is one of the most vulnerable organs to oxidative stress due to its specific structure and function [25]. It is clear that the production of reactive oxygen species (ROS) during both ischemia and reperfusion is a major factor contributing to IR-induced lung injury [2,3,21]. 

The first product of lipid peroxidation, LOOH, decomposes into aldehydes including MDA. MDA is a highly toxic molecule that rapidly interacts with DNA and proteins and is often referred to as mutagenic [7]. In the present study, the severely accumulated lipid peroxidation products, LOOH and MDA levels, indicated increased formation of ROS in both lung tissue and BALF, as well as in serum in the IR group.

These reactive aldehydes accelerate cell death by disrupting the normal cellular function through reactions with proteins, nucleic acids, and amino-phospholipids. Gulmen et al. found that levels of MDA in lung tissue and BALF significantly increased after aortic IR [26]. Therefore, MDA is regarded as an indirect indicator of the formation of oxygen free radicals and reactive oxygen species.

Many enzymes such as SOD and reduced GSH may exert protective effects against the toxic oxygen metabolites in lung tissue [27]. In our study, low SOD and GSH activities in the aortic IR group demonstrated that endogenous SOD and GSH were consumed, which causes an oxidative defensive response. Serum [20], BALF, and lung tissue [2] SOD and GSH levels were found decreased in our previous studies and in other similar studies with an experimental lung injury model [28].

In our results, in accordance with the increase in lipid peroxidation products and decrease in antioxidant enzyme levels, PAB levels were increased and FRAP levels were decreased in serum, BALF, and lung tissue samples in the IR group. These findings suggest that the pro-oxidant/antioxidant balance changed in favor of pro-oxidants.

In the present study, the administration of Tβ4 before ischemia and before reperfusion significantly decreased the concentration of LOOH and MDA. This finding is an important indicator for the capability of Tβ4 in the prevention of ALI formation, since oxidative stress is a key factor in ALI development.

On the other hand, in accordance with the decrease in PAB and increase in FRAP, the increase in both SOD and GSH levels in serum, BALF, and lung tissue samples suggests that the pro-oxidant/antioxidant balance changed in favor of antioxidants following treatment with Tβ4.

Furthermore, Tβ4 was shown to elicit a protective effect against cell death by increasing the expression of antioxidant enzymes, by decreasing formation of superoxide radicals, and by increasing the membrane potential of mitochondria against oxidative stress [8]. These results also support our findings. Many studies like ours revealed the antioxidant effect of Tβ4. However, the underlying mechanism of this effect is not completely understood.

Aortic IR injury induces systemic effects in the lung, kidney, heart, and liver, and it is characterized by neutrophil sequestration and the release of significant amounts of ROS into circulation [29]. The increase in ROS triggers the expression of several pro-inflammatory genes, and this phenomenon is called post-ischemic inflammation, which is another factor responsible for the formation of ALI [7,30]. ROS can attack alveolar capillary membranes causing changes in the lung permeability and an increase in extravascular lung water, resulting in lung edema [2]. High-permeability pulmonary edema is a hallmark feature of ALI. In the present study, the lung W/D ratio was significantly increased in the IR group compared with control, which indicates edema formation. In addition, the increase in oxidants and inflammatory mediators in BALF samples of the IR group indicates alveolar capillary membrane disruption.

In the clinical and experimental models of IR, various pro-inflammatory cytokines like TNF-α, IL-1β, and IL-6 were suggested to be responsible for ALI development [4]. In our study, we found that serum, BALF, and lung tissue TNF-α, IL-1β, and IL-6 levels were increased in aortic IR induced lung injury.

TNF-α was shown to play a key role in lung reperfusion injury, neutrophil activation, and infiltration to the lungs [31]. TNF-α was reported to cause direct mitochondrial toxicity and to induce apoptotic and necrotic cell death. 

Alveolar epithelial cells are usually identified as targets of inflammatory cells [32]. Activated neutrophils cause the release of free radicals, proteolytic enzymes, and peroxidases. Therefore, an increase in pulmonary vascular permeability accompanies ALI [33].

On the other hand, TNF-α was reported to be a strong stimulant of inducible nitric oxide synthase (iNOS) and may cause a large amount of nitric oxide (NO) production. Several studies pointed out that the production of NO in high quantity causes inflammation, as well as nitrosative stress [34]. 

IL-6 is accepted as an early biomarker of tissue damage, and it is thought to be the key pro-inflammatory cytokine for accumulation of neutrophils in the lung. IL-6 levels were found to be significantly elevated in patients going under abdominal aortic aneurism (AAA) surgery [33]. TNF-α and IL-6 stimulate further liberalization of cytokines, which leads to classic symptoms of inflammation [6]. 

IL-1 β levels were shown to increase in lung injury caused by lower-extremity IR, and it is referred to as an important mediator in local and interorgan IR [30].

In the present study, the increase in TNF-α, IL-1β, and IL-6 levels in BALF samples of the IR group also indicates alveolar capillary membrane disruption.

Furthermore, these indicated results support our histological findings of interstitial edema formation and neutrophil infiltration in the alveolar area of the experimental group of rats (Figure 1). According to our histological findings, characteristic morphologic changes like capillary congestion in pulmonary capillaries, interstitial edema, infiltration. and intra-alveolar hemorrhages were observed in IR group rats. However, treatment with Tβ4, either before ischemia or prior to reperfusion, led to a significant histological improvement of the lung tissue, recovering the morphologic changes and damage caused by IR (Figure 1). Those findings suggest that Tβ4 may prevent free-radical formation and cytokine-mediated acute inflammation. 

On the other hand, the increase in the production of ROS was reported to activate the transcription factor NF-κB [8]. In addition, TNF-α and IL-1β are considered to be cytokines that activate NF-κB. NF-κB is an important transcription factor that upregulates gene expression of various pro-inflammatory cytokines including TNF-α, IL-1β, and IL-6 [35] chemokines, growth factors, and cell-surface adhesion molecules and apoptosis signals which play an important role in the pathogenesis of ALI. Qiu et al. [36] explored whether Tβ4 inhibits TNF-α-induced NF-κB activation. Sosne et al. [37] also found that Tβ4 suppression of TNF-α-induced NF-κB activation is not immediate, as maximal effects of Tβ4 were time-related. Also, NF-κB was reported to be associated with initiation of neutrophil infiltration to lungs, increased epithelial permeability, and lipid peroxidation in animal models [35].

According to our findings, the increase of NF-κB in all samples after IR is parallel to the increases of pro-inflammatory cytokines. Therefore, we may say that the NF-κB-mediated upregulation of pro-inflammatory cytokines is responsible for the inflammation-mediated ALI. On the other hand, both pre-ischemia and pre-reperfusion groups of rats treated with Tβ4 showed decreased pro-inflammatory cytokine levels. These findings clearly indicate that Tβ4 prevents inflammation-mediated ALI via NF-κB deactivation and, therefore, decreasing pro-inflammatory cytokine levels. Crockford et al. suggested that Tβ4 causes its anti-inflammatory effect by suppressing the activation and translocation of NF-κB [9]. These studies support our results.

While these results are all in favor of Tβ4, there are studies where Tβ4 was not found to be effective, such as that by Stark et al. [38]. They studied a cardiopulmonary bypass model in a pig and investigated whether the myocardial ischemia–reperfusion injury could be attenuated by Tβ4 application. While they did not find promising results in their study, this was very important as it was one of the few in vivo studies carried out on larger animals.

## 5. Conclusions

Finally, to sum up our findings, we may conclude that both pre-ischemia and pre-reperfusion treatment with Tβ4 improves impaired redox homeostasis and the inflammatory process caused by aortic IR-induced lung injury in rats. 

Our study clearly demonstrated that administrating Tβ4 not only reduced lung oxidative stress and inflammation following infrarenal abdominal aortic IR model, but also prevented lung tissue injury. We propose that the mechanism underlying this protective effect of Tβ4 for improving lung injury involves the reduction of oxidative stress and subsequent lipid peroxidation, and the inhibition of inflammatory response by reducing the levels of pro-inflammatory cytokines and NF-κB. Administration of Tβ4 before surgery can be useful for preventing the development of inflammation and/or oxidative stress caused by IR injury.

Nevertheless, further experimental studies are required to specifically define the precise mechanism of antioxidant and anti-inflammatory effects of Tβ4 on a molecular basis. In this way, the formation of remote organ failure could be prevented to some extent.

## Figures and Tables

**Table 1:** The results of serum, bronchoalveolar lavage fluid (BALF), and lung tissue oxidative stress parameters in indicated groups.

		Groups			
		Control *n* = 8	IR *n* = 8	Tβ4 + IR *n* = 8	I+Tβ4+R *n* = 8
Serum Oxidant /Anti-oxidant levels	LOOH (nmol/L)	0.42 ± 0.02	0.30 ± 0.02 ***	0.35 ± 0.01 ^≠^	0.32 ± 0.02 ^≠≠≠^
	MDA (µmol/mL)	18.11 ± 0.72	8.49 ± 0.36 ***	10.33 ± 1.01 ^≠≠≠^	8.84 ± 0.48 ^≠≠≠^
	GSH ^†^ (µmol/g Hb)	0.84 ± 0.01	1.19 ± 0.06 ***	1.02 ± 0.06 ^≠^	1.07 ± 0.03 ^≠≠≠^
	SOD (U/L)	17.95 ± 1.50	10.81 ± 0.74 ***	16.30 ± 0.70 ^≠≠^	17.42 ± 1.24 ^≠≠^
	PAB (H_2_O_2_ %)	24.37 ± 1.07	41.87 ± 2.37 ***	26.58 ± 1.72 ^≠≠≠^	25.23 ± 2.24 ^≠≠≠^
	FRAP (mmol uric acid)	0.04 ± 0.00	0.08 ± 0.01 ***	0.06 ± 0.01 ^≠^	0.07 ± 0.01 ^≠≠^
BALF Oxidant /Anti-oxidant levels	LOOH (nmol/L)	4.08 ± 0.22	5.26 ± 0.13 ***	4.30 ± 0.17 ^≠≠^	4.28 ± 0.14 ^≠≠^
	MDA (µmol/mL)	13.76 ± 0.24	19.98 ± 1.53 ***	14.59 ± 0.32 ^≠≠≠^	14.42 ± 0.36 ^≠≠≠^
	GSH (µmol/L)	0.69 ± 0.02	0.25 ± 0.02 ***	0.60 ± 0.05 ^≠≠≠^	0.63 ± 0.02 ^≠≠≠^
	SOD (U/L)	19.50 ± 1.95	10.71 ± 0.86 ***	16.84 ± 1.00 ^≠^	17.91 ± 1.03 ^≠≠^
	PAB (H_2_O_2_ %)	36.69 ± 0.82	43.34 ± 0.58 ***	37.75 ± 1.30 ^≠≠^	37.49 ± 1.00 ^≠≠^
	FRAP (mmol uric acid)	0.06 ± 0.00	0.02 ± 0.01 ***	0.04 ± 0.01 ^≠^	0.05 ± 0.01 ^≠≠^
Tissue Oxidant /Anti-oxidant levels	LOOH (nmol/wet tissue)	3.59 ± 0.03	4.36 ± 0.05 ***	3.76 ± 0.07 ^≠≠≠^	3.76 ± 0.02 ^≠≠≠^
	MDA (µmol/wet tissue)	65.79 ± 0.68	84.09 ± 1.72 ***	69.42 ± 1.09 ^≠≠≠^	69.54 ± 0.37 ^≠≠≠^
	GSH (µmol/wet tissue)	0.34 ± 0.03	0.17 ± 0.02 ***	0.28 ± 0.02 ^≠≠^	0.30 ± 0.01 ^≠≠≠^
	SOD (U/wet tissue)	20.77 ± 1.62	13.39 ± 0.93 **	18.79 ± 1.28 ^≠^	20.64 ± 0.92 ^≠≠^
	PAB (H_2_O_2_ % /wet tissue)	113.40 ± 0.47	135.20± 2.33 ***	120.60± 1.63 ^≠≠≠^	118.00± 2.39 ^≠≠≠^
	FRAP (mmol uric acid /wet tissue)	1.53 ± 0.03	1.01 ± 0.03 ***	1.38 ± 0.01 ^≠≠≠^	1.45 ± 0.01 ^≠≠≠^

LOOH: lipid hydroperoxide, MDA: malondialdehyde, PAB: pro-oxidant/anti-oxidant balance, Cu,Zn-SOD: superoxide dismutase, GSH: glutathione, FRAP: ferric reducing antioxidant power (** *p* < 0.01, *** *p* < 0.001 vs. control; ≠ *p* < 0.05, ≠≠ *p* < 0.01, ≠≠≠ *p* < 0.001 vs. IR). † Erythrocyte GSH levels.

**Table 2:** The results of serum, BALF, and lung tissue pro-inflammatory cytokines and nuclear factor kappa B (NF-κB) in indicated groups.

		Groups			
		Control *n* = 8	IR *n* = 8	Tβ4 + IR *n* = 8	I+Tβ4+R *n* = 8
Serum pro-inflammatory cytokines and NF-κB levels	TNF-α (pg/mL)	45.36 ± 1.43	79.56 ± 3.18 ***	50.46 ± 1.17 ^≠≠≠^	45.49 ± 1.14 ^≠≠≠^
	IL-6 (pg/mL)	1517.00 ± 49.73	2448.00 ± 60.83 ***	1561.00 ± 54.31 ^≠≠≠^	1555.00 ± 44.22 ^≠≠≠^
	IL-1β (pg/mL)	42.79 ± 3.19	81.82 ± 2.58 ***	50.11 ± 3.32 ^≠≠≠^	46.32 ± 2.22 ^≠≠≠^
	NF-κB (ng/mL)	6.00 ± 0.52	12.05 ± 1.30 ***	7.10 ± 0.25 ^≠≠≠^	6.74 ± 0.25 ^≠≠≠^
BALF pro-inflammatory cytokines and NF-κB levels	TNF-α (pg/mL)	45.74 ± 1.63	78.58 ± 5.54 ***	53.95 ± 2.77 ^≠≠≠^	51.35 ± 1.66 ^≠≠≠^
	IL-6 (pg/mL)	1478.00 ± 52.67	2630.00 ± 71.86 ***	1607.00 ± 70.06 ^≠≠≠^	1541.00 ± 47.37 ^≠≠≠^
	IL-1β (pg/mL)	233.90 ± 10.33	411.60 ± 27.90 ***	247.00 ± 19.54 ^≠≠≠^	227.10 ± 11.80 ^≠≠≠^
	NF-κB (ng/mL)	9.63 ± 0.35	17.56 ± 1.88 ***	10.25 ± 0.48 ^≠≠≠^	9.78 ± 0.45 ^≠≠≠^
Tissue pro-inflammatory cytokines and NF-κB levels	TNF-α (pg/100 µg protein)	44.80 ± 1.01	72.32 ± 4.01 ***	46.57 ± 1.02 ^≠≠≠^	42.59 ± 1.19 ^≠≠≠^
	IL-6 (pg/100 µg protein)	109.30 ± 1.63	164.40 ± 3.07 ***	117.60 ± 2.25 ^≠≠≠^	112.60 ± 1.94 ^≠≠≠^
	IL-1β (pg/100 µg protein)	130.50 ± 7.63	190.00 ± 7.14 ***	152.20 ± 3.69 ^≠≠^	130.50 ± 7.81 ^≠≠≠^
	NF-κB (ng/100 µg protein)	1.00 ± 0.04	1.50 ± 0.06 ***	1.17 ± 0.03 ^≠≠≠^	1.05 ± 0.06 ^≠≠≠^

TNF-α: tumor necrosis factor alpha, IL-1β: Interleukin 1β, IL-6: Interleukin 6, NF-κB: nuclear factor-kappa B (*** *p* < 0.001 vs. control; ≠≠ *p* < 0.01, ≠≠≠ *p* < 0.001 vs. IR).
